# Microbiota alteration is associated with the development of stress-induced despair behavior

**DOI:** 10.1038/srep43859

**Published:** 2017-03-07

**Authors:** Ioana A. Marin, Jennifer E. Goertz, Tiantian Ren, Stephen S. Rich, Suna Onengut-Gumuscu, Emily Farber, Martin Wu, Christopher C. Overall, Jonathan Kipnis, Alban Gaultier

**Affiliations:** 1 Center for Brain Immunology and Glia, University of Virginia, Charlottesville, VA, 22904, USA; 2 Department of Neuroscience, University of Virginia, Charlottesville, VA, 22904, USA; 3 Graduate Program in Neuroscience, School of Medicine, University of Virginia, Charlottesville, VA, 22908, USA; 4 Department of Biology, University of Virginia, Charlottesville, VA, 22904, USA; 5 Center for Public Health Genomics, University of Virginia, Charlottesville, VA, 22908, USA.

## Abstract

Depressive disorders often run in families, which, in addition to the genetic component, may point to the microbiome as a causative agent. Here, we employed a combination of behavioral, molecular and computational techniques to test the role of the microbiota in mediating despair behavior. In chronically stressed mice displaying despair behavior, we found that the microbiota composition and the metabolic signature dramatically change. Specifically, we observed reduced *Lactobacillus* and increased circulating kynurenine levels as the most prominent changes in stressed mice. Restoring intestinal *Lactobacillus* levels was sufficient to improve the metabolic alterations and behavioral abnormalities. Mechanistically, we identified that *Lactobacillus*-derived reactive oxygen species may suppress host kynurenine metabolism, by inhibiting the expression of the metabolizing enzyme, IDO1, in the intestine. Moreover, maintaining elevated kynurenine levels during *Lactobacillus* supplementation diminished the treatment benefits. Collectively, our data provide a mechanistic scenario for how a microbiota player (*Lactobacillus*) may contribute to regulating metabolism and resilience during stress.

Depression is one of the most common types of mental illnesses, affecting up to 7% of the population^1^,^2^. While improved diagnosis led to appreciation of the frequency of the disorder, better understanding of the mechanisms leading to depression is needed for the development of new therapeutic approaches for this debilitating disease. Based on both human and animal studies, several hypotheses have been proposed to underlie the etiology of depression, including monoamine deficiency, stress response dysregulation, neuronal plasticity deficits, and inflammation^1^,^2^,^3^. Genetic polymorphisms have also been linked to an increased risk for developing depression^2^. However, their prevalence cannot account for the frequency and familial nature of the disorder. Family history can also be viewed as having a “shared environment”, i.e. a shared microbiota composition. Therefore, we decided to explore the role of the gut microbiota in the development and maintenance of depressive behavior.

The microbiota, the collection of organisms inhabiting various organs, has been increasingly recognized to modulate host physiology^4^. Changes in gut microbiota composition have been linked to CNS function in animal models and human studies. In most studies, germ free mice show a decreased anxiety phenotype, but an exacerbated response to stress, and have numerous neurochemical disturbances^5^,^6^,^7^,^8^,^9^. Alteration of gut microbiota composition in despair and anxiety disorders has long been suspected from clinical observations, such as high co-morbidities of colitis and depression^10^,^11^,^12^.

In this study, we set out to understand whether and how microbiota alterations contribute to CNS dysfunction and behavioral abnormalities and whether the gut microbiota could be therapeutically targeted to alleviate depression. Here, we show that chronic stress significantly alters intestinal microbiota composition, primarily depleting the *Lactobacillus* compartment. ROS produced by *Lactobacilli* can inhibit kynurenine metabolism, a pathway that can negatively impact the brain when dysregulated. Surprisingly, therapeutic administration of *L. reuteri* to stressed mice improves metabolic homeostasis and corrects stress-induced despair behaviors.

## Results

### Microbiota composition is altered by unpredictable chronic mild stress

**Figure 1:**
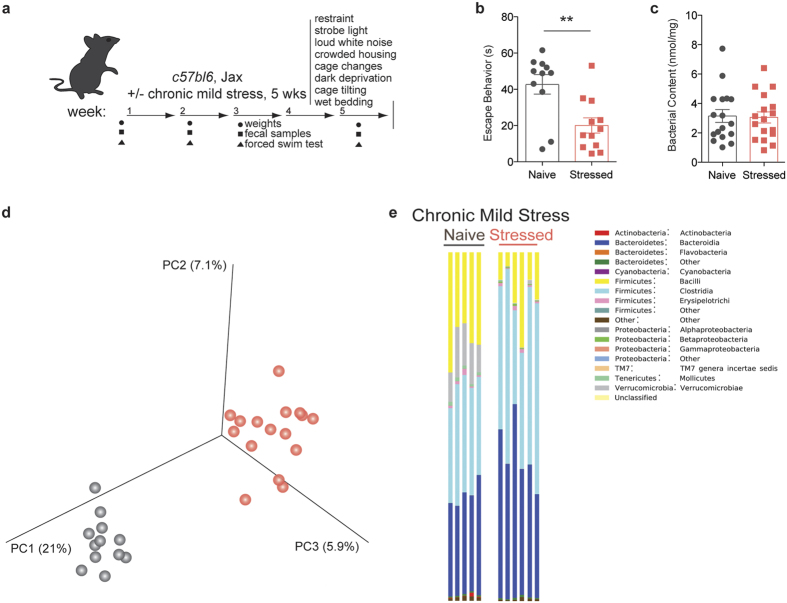
Unpredictable chronic mild stress (UCMS) induces despair behavior and microbiota dysregulation. (**a**) Experimental design. (**b**) Quantification of escape behavior in the forced swim test (n = 11 naïve and 12 stressed; representative of 3 independent experiments; two-tailed t-test with Welch’s correction, **p < 0.01; mean ± s.e.m.). (**c**) Total bacterial load quantification by qRT-PCR of 16S rRNA (n = 17 samples per group; two-tailed t-test; mean ± s.e.m.). (**d**) Principal coordinate analysis of microbiome communities in naïve and stressed mice. Analysis based on 2 UCMS experiments (n = 12 naïve and 16 stressed; representative of 2 sequencing experiments) (**e**) Representative graphs of bacterial class distribution in individual subjects show a decrease in bacilli (yellow) (n = 5 naïve and 6 stressed; representative of 2 sequencing experiments).

To determine whether chronic stress can directly affect the microbiota, we chose the unpredictable chronic mild stress (UCMS) model to induce despair behavior^13^,^14^. Considering the dynamic nature of the intestinal microbiota, we posited that lasting changes must be observed over a prolonged period of time (e.g. weeks-months). The UCMS model seemed particularly appropriate due to the length and variety of the stress protocol (Fig. 1a). Consistent with previous reports, this protocol effectively induced despair behavior, as measured by the forced swim test (t(19) = 3.343, Welch’s correction applied, p = 0.0034; Fig. 1b)^3^,^15^,^16^. The assay measures the amount of time an animal struggles to escape an uncomfortable situation, a behavior typically affected in most models of depression and corrected by anti-depressant treatment. We verified that the forced swim test results were true despair behavior, as the animals show normal activity and locomotion in the open field test (Sup. Fig. 1a,b). The UCMS protocol did not significantly impact the weight and the food intake of stressed mice when compared to the control group (Sup. Fig. 1c,d).

**Figure 2:**
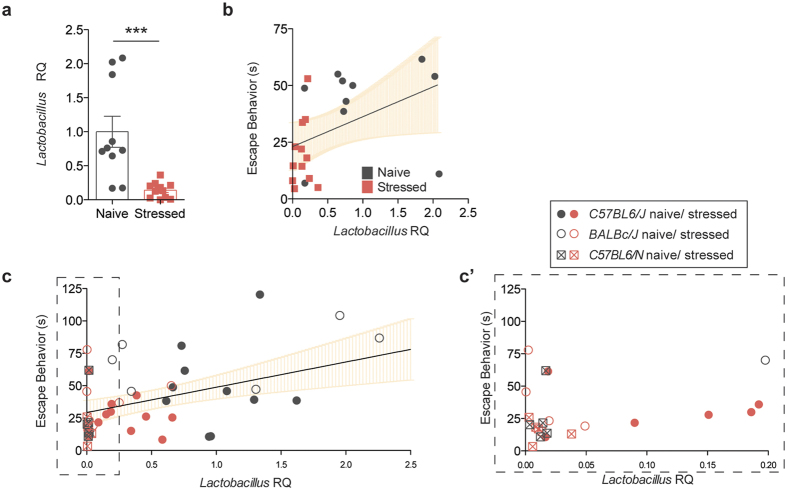
*Lactobacillus* levels correlate with depressive behavior. (**a**) *Lactobacillus* quantification in fecal samples by qRT-PCR, relative to 16S rRNA (n = 10 naïve and 11 stressed; representative of 3 independent experiments; two-tailed t-test with Welch’s correction, ***p < 0.001; mean ± s.e.m.). (**b**) Correlation analysis between *Lactobacillus* levels and escape behavior (n = 22 pairs, two-tailed Spearman r, **p = 0.01, line of best fit with 95% CI). (**c**) Correlation analysis between *Lactobacillus* levels and escape behavior in C57BL/6J (Jax), BALB/cJ (Jax), and C57BL/6N (Taconic), naive and stressed (n = 45 pairs, two-tailed Spearman r, **p = 0.01, line of best fit with 95% CI). (**c**’) Dashed insert with expanded X-axis for better resolution.

In order to assess the changes in microbiota composition that occur during chronic stress, we performed 16S rRNA sequencing on genomic DNA isolated from the fecal samples of naïve and stressed mice. The quantity of bacterial DNA in fecal pellets was not affected by stress, as demonstrated by 16S qPCR (t(33) = 0.4447, p = 6594; Fig. 1c). In terms of microbiota composition, principal coordinate analysis shows distinct clustering between samples from naïve and stressed mice, indicative of differences between the groups (Fig. 1d). A more in-depth taxonomic analysis of bacterial types revealed several changes in the microbiota composition (Fig. 1e shows one experimental cohort, Sup. Fig. 2 shows a different experimental cohort; bacterial classes are shown for ease of visualization). In our sequencing runs we observed between 14 and 29 significantly different genera between the naïve and stressed conditions. The variability in the starting microbiota (of naïve mice) and its changes (after stress) is not unexpected, as different shipments of mice, even from the same vendor, can have different microbiota compositions^17^,^18^. Overall, the most conserved microbiota change across all independent experiments was a decrease in *bacillus* class members in stressed mice (Fig. 1e, Sup. Fig. 2a). This class encompasses *Lactobacillus* and *Turicibacter*, and the decrease was also detectable at the genus level (Sup. Fig. 2b). Due to the abundance of literature linking *Lactobacilli* and behavior and the lack of studies and tools regarding *Turicibacter* species, we further focused on *Lactobacillus* as a confident potential player in the despair phenotype. We verified the net loss of *Lactobacilli* by qPCR (t(19) = 4.103, Welch’s correction applied, p = 0.0006; Fig. 2a) and selective fecal sample cultures using MRS agar supplemented with azide (t(9) = 2.993, Welch’s correction applied, p = 0.0157; Sup. Fig. 3a,b)^19^. These results demonstrate that chronic stress disturbs the microbiota homeostasis, in particular by decreasing the *Lactobacillus* levels. Correlation analysis returned a positive correlation (Spearman r = 0.5246, p = 0.0122) between the relative *Lactobacillus* load and the escape behavior displayed by a mouse (Fig. 2b). Our observation was not limited to C57BL/6J, as BALB/cJ and C57BL/6N mice also show significant correlation (Spearman r = 0.4682, p = 0.0012) between *Lactobacillus* levels and their escape behavior (Fig. 2c). Interestingly, C57BL/6N mice had very low starting levels of *Lactobacillus*, which corresponded to low escape behavior even in the absence of stress. Our data are in agreement with recent studies showing associations between lower *Lactobacillus* levels and stress^20^,^21^.

To gain insight into potential causes for changed microbiota composition, we further characterized intestinal physiology and immunity. Similarly to previous reports using stress models^22^,^23^, large intestinal transit time was significantly decreased in the stressed animals (t(19) = 4.275, Welch’s correction applied, p = 0.0004; Sup. Fig. 4a). Furthermore, we observed an increase in the total size and cellular content of the stressed small intestines (t(22) = 3.574, p = 0.0017; t(22) = 2.248, p = 0.0349; Sup. Fig. 4b,c). These changes in intestinal physiology in response to stress may underlie microbiota changes.

### Treatment with a *Lactobacillus* species ameliorates despair behavior by restoring kynurenine metabolism

**Figure 3:**
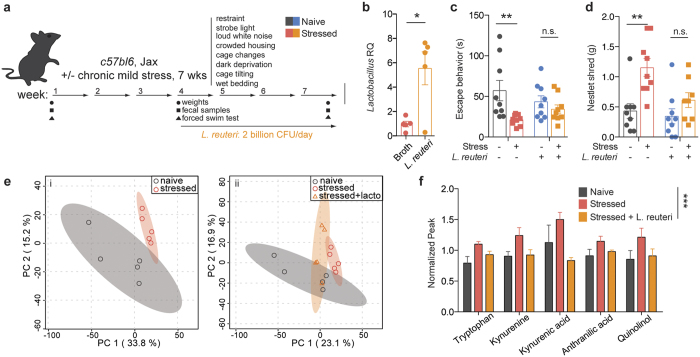
Treatment with probiotic *L. reuteri* ameliorates the escape behavior induced by chronic stress. (**a**) Experimental design of *L. reuteri* supplementation regimen. (**b**) qRT-PCR quantification of *Lactobacillus* levels in fecal samples of *L. reuteri* or broth-control treated mice, relative to 16S rRNA (n = 5; representative of 2 independent experiments; two-tailed t-test, *p < 0.05; mean ± s.e.m.). (**c**) Forced swim test quantification of escape behavior of naïve and stressed mice treated with either *L. reuteri* or bacteria-free broth (n = 9 per group; representative of 2 independent experiments; 2-way ANOVA followed by Bonferroni post-hoc, **p < 0.01; mean ± s.e.m.). (**d**) Nestlet shredding test quantification of escape behavior of naïve and stressed mice treated with either *L. reuteri* or bacteria-free broth (n = 9 per group; representative of 2 independent experiments; 2-way ANOVA followed by Bonferroni post-hoc, **p < 0.01; mean ± s.e.m.). (**e**) Principal component analyses of serum metabolite composition after untargeted metabolomics assay (n = 5 mice per group) showing two (i) or three (ii) group comparisons; shaded areas represent 95% CI. (**f**) Normalized MS peaks of tryptophan - kynurenine pathway metabolites in the sera of naïve, stressed and *L. reuteri* treated stressed mice (n = 3–5 per group; 2-way ANOVA, ***p < 0.001; mean ± s.e.m.).

To assess whether *Lactobacillus* levels may play a role in mediating despair behaviors, we attempted to replenish the levels of the bacteria and then measure escape behavior. To this end, we subjected mice to the UCMS protocol for three weeks and then supplemented their diet with live cultures of *L. reuteri*, while continuing the stress protocol for additional 4 weeks (Fig. 3a). *Lactobacillus reuteri* (ATCC 23272) is a species that colonizes several vertebrate hosts, including rodents and humans, and was shown to improve despair and anxiety-like behaviors, including the forced swim test in mice^24^,^25^. This regimen indeed elevated *Lactobacillus* levels in the stressed mice (Fig. 3b, t(8) = 3.330, p = 0.0104). The increase in overall *Lactobacillus* was not due to a re-expansion of endogenous bacteria, but due to *L. reuteri* supplementation (Sup. Fig. 5). Moreover, in our experimental paradigm, *L. reuteri* supplementation ameliorated the despair behavior induced by UCMS (Fig. 3c, F_stress_(1, 32) = 8.569, p = 0.0062; F_interaction_(1, 32) = 3.005, p = 0.0926; Bonferroni post-hoc t_broth_ = 3.96, t_*L.reuteri*_ = 0.8441). *L. reuteri* also improved the compulsive behavior observed in stressed mice, as measured by the nestlet shredding test (Fig. 3d, F_stress_(1, 31) = 13.7, p = 0.0008; F_treatment_(1, 31) = 5.508, p = 0.0255; Bonferroni post-hoc t_broth_ = 3.861, t_*L.reuteri*_ = 1.412). These data indicate that *Lactobacillus* levels may be mediating, at least in part, the depressive-like behavior.

**Figure 4:**
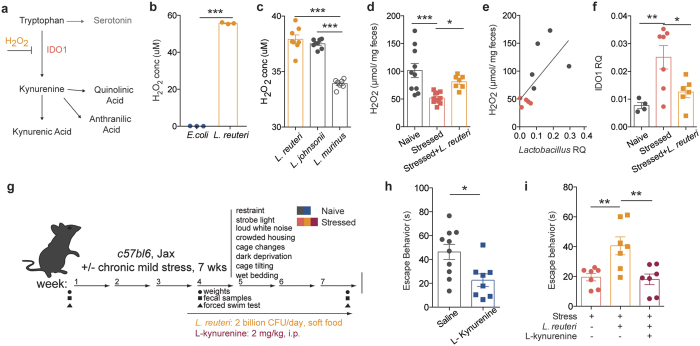
*Lactobacillus* supplementation improves behavior by moderating kynurenine metabolism. (**a**) Representation of tryptophan-kynurenine pathway, depicting H_2_O_2_ inhibition of pathway-initiating enzyme IDO1. (**b**) Production of H_2_O_2_ by E. coli and *L. reuteri* (n = 3 wells per group; representative of 2 independent experiments; two-tailed t-test, ***p < 0.001; mean ± s.e.m.). (**c**) Production of H_2_O_2_ by endogenous *Lactobacillus* species compared to *L. reuteri* (n = 7–8 colonies per group; one-way ANOVA with Dunnet’s multiple comparisons, ***p < 0.001; mean ± s.e.m.). (**d**) Fecal H_2_O_2_ levels in naïve, stressed and *L. reuteri* treated stressed mice (n = 10 naïve, 11 stressed and 7 stressed + *L.reuteri* mice per group; one-way ANOVA with Dunnet’s multiple comparisons, *p < 0.05, ***p < 0.001; mean ± s.e.m.). (**e**) Correlation between *Lactobacillus* and H_2_O_2_ levels in naïve and stressed mice (n = 5 mice per group; Spearman r test, **p = 0.01). (**f**) qRT-PCR quantification of *ido1* expression in the intestines of naïve, stressed mice and *L. reuteri* treated stressed mice, relative to GAPDH. (n = 4 naïve, 7 stressed and stressed + *L.reuteri* mice per group, 1-way ANOVA followed by Dunnett’s post-hoc, p < 0.01; mean ± s.e.m.). (**g**) Experimental design of *L. reuteri* and/or kynurenine administration. (**h**) Forced swim test quantification of escape behavior of naïve mice treated with L-kynurenine or saline control (n = 10 saline and 8 L-kynurenine mice per group; representative of 2 independent experiments; two-tailed t test with Welch’s correction, *p < 0.05; mean ± s.e.m.). (**i**) Forced swim test quantification of escape behavior of stressed mice treated with either *L. reuteri* alone or *L. reuteri* and L-kynurenine (n = 7 per group; one-way ANOVA followed by Dunnett’s post-hoc, **p < 0.01; mean ± s.e.m.).

To get an insight into the potential mechanism of *Lactobacillus*-supported resiliency, we performed untargeted metabolomics analysis of serum samples to identify if and how metabolites composition was altered after chronic stress. Principal component analysis showed that the metabolic profile of stressed mice is clustered distinctly from that of naïve mice (Fig. 3ei). Treatment with *L. reuteri* modified the metabolic profile of stressed mice to an intermediate profile, suggesting that some of the stress associated metabolic alterations may be a consequence of decreased *Lactobacillus* levels, while others may be a direct effect of *L. reuteri* administration (Fig. 3eii). While several molecules were significantly different in our analysis (234 out of 4900 spectra), most returned spectra were not confidently matched to known molecules due to limitations in the metabolite library (Sup. Fig. 6). Of the identified compounds, we mined those increased in stressed animals and normalized by *L. reuteri* treatment. Among them, metabolites in the tryptophan-kynurenine pathway presented this pattern (Fig. 3f, F(2, 53) = 12.13, p < 0.0001). Evidence of dysregulation of this pathway in depressed patients, as well as its recently described role in the initiation of despair behaviors^26^, have made the tryptophan-kynurenine pathway particularly compelling as part of the potential mechanism mediating the microbiome effects on behavior. IDO1 is the main enzyme metabolizing L-tryptophan to kynurenine outside of the liver (where TDO is the primary enzyme)^27^. The effects of *Lactobacillus* strains on *ido1* expression remain unclear. While it appears that in the context of inflammatory disease several *Lactobacillus* strains can increase IDO1 expression or activity^28^,^29^, in other models *Lactobacillus* administration dampens it^30^,^31^. Intriguingly, a recent study^32^ showed that, at steady state, *Lactobacillus* administration can directly modulate kynurenine metabolism, by inhibiting the pathway initiating enzyme IDO1 via production of reactive oxygen species (i.e. H_2_O_2_, summarized in Fig. 4a).

To further explore this question in our study, we verified that cultured *L. reuteri* produced a significant amount of H_2_O_2_ *in vitro*, when compared to *E.coli*, a negative control that does not produce H_2_O_2_ (Fig. 4b, t(4) = 208.2, p < 0.0001). In addition, we also compared the ROS production from *L. reuteri* with that of endogenous *Lactobacillus* in Jackson C57BL6 mice, *L. johnsonii*, and observed they were comparable (Fig. 4c and Sup. Fig. 3c, Tuckey’s posthoc q = 1.592, p = 0.51). *L. murinus*, the endogenous *Lactobacillus* found in Taconic C57BL6/N mice was also included for comparison. We next measured peroxide levels in the fecal contents of the stressed mice and discovered that H_2_O_2_ levels were decreased in the stressed mice; more importantly, and in agreement with our hypothesis, therapeutic administration of *L. reuteri* significantly raised the level of H_2_O_2_ *in vivo* (F(2, 25) = 9.907, p = 0.0007, Fig. 4d). Moreover, we observed a significant correlation between the drop in *Lactobacillus* levels and the levels of H_2_O_2_ (Fig. 4e, Spearman r = 0.7818, p = 0.0105). We further verified the kynurenine pathway dysregulation by probing for *ido1* mRNA in the intestine. Our results show increased *ido1* expression in the intestines after stress, which is decreased after *L. reuteri* treatment (F_stress_(2, 14) = 7.274, p = 0.0068), in accordance to the effects observed on ROS production (Fig. 4f). To further investigate the causative role of kynurenine metabolism in mediating despair behavior we treated naïve mice with L-kynurenine daily (i.p., Fig. 4g) and observed a significant reduction in escape behavior (t(16) = 2.861, Welch’s correction applied, p = 0.0113) at the end of the 4-week protocol (Fig. 4h). Moreover, in order to show that the benefit of *Lactobacillus* supplementation is by reducing kynurenine level, we treated stressed mice simultaneously with *L.reuteri* and L-kynurenine, expecting kynurenine to bypass the benefits of *L.reuteri* supplementation. Indeed, while *L. reuteri* alone increased the escape behavior of stressed mice, kynurenine administration abrogated the beneficial effect of *L.reuteri* (F(2, 18) = 8.632, p = 0.0024; Fig. 4i), even with elevated levels of *Lactobacillus* and H_2_O_2_ (Sup. Fig. 7a,b).

## Discussion

Taken together, our results demonstrate that microbiome homeostasis was robustly altered in animals undergoing UCMS, with a consistent decrease in *Lactobacilli*. This finding was shared across three strains of mice (C57BL/6J, as BALB/cJ and C57BL/6N). Moreover, our data suggest that the production of H_2_O_2_ by *Lactobacillus* may be protective against the development of despair behavior by direct inhibition of intestinal *ido1* expression and decrease in the circulating level of kynurenine, a metabolite associated with depression^26^.

Our results are in agreement with recent literature demonstrating that microbiome composition is modified with acute and chronic stress^20^,^33^,^34^. Microbiome dysbiosis is also detected in humans affected by major depressive disorders and the transplantation of the biota from these patients in germ free mice can induce despair behavior^9^. Beyond describing microbiome fluctuation as a consequence of UCMS, we further demonstrated that levels of *Lactobacillus* correlate with the susceptibility to and severity of despair behaviors. Indeed, animals exhibiting low (i.e. Taconic C57BL/6N mice) intestinal *Lactobacillus* levels present with a basal despair phenotype, when compared to animals with higher levels of *Lactobacillus* (i.e. Jackson C57BL/6J mice). Accordingly, therapeutic administration of a probiotic *Lactobacillus* species during UCMS was sufficient to improve the despair symptoms. Further works will be needed to explore the role of other populations of bacteria affected by UCMS, as well as *Lactobacillus* strain differences and their abilities to improve behavior.

Recently, members of the *Lactobacillus* genus have been shown to affect a multitude of aspects of human physiology, as they colonize several sites of the body, including the skin, the vagina, and the entirety of the gastrointestinal tract, starting with the oral cavity^35^. Perhaps best studied in the vagina, *Lactobacilli* protect against infection by producing a diversity of antimicrobial factors, including lactic acid, peroxide, bacteriocins, as well as by resource competition^35^,^36^,^37^. Although in a few contexts increased levels of *Lactobacilli* are associated with pathology, e.g. dental cavities^38^, the bacteria are largely non-pathogenic or beneficial. From dysbioses or probiotic studies, *Lactobacilli* are associated with protection against infection, improved recovery after enteric infections, decreased colitis pathology, and better cognitive function^25^,^39^,^40^,^41^.

While *Lactobacilli* are able to control other microbial communities through secretion of antimicrobial factors, genetic limitations make them more sensitive to environmental conditions. In particular, many *Lactobacillus* genus members are unable to synthesize amino acids and purines and thus rely on nutrient rich environments and other bacteria for supply of essential building blocks^42^,^43^,^44^,^45^. We hypothesize that, in the context of a faster intestinal transit, such as the one observed in stressed animals, fluctuating availability of nutrients and symbiotic bacteria will impact the renewal of the *Lactobacillus* niche^46^. Further studies will be able to determine whether there is indeed a causal relationship between increased intestinal motility and microbiota alteration in the context of stress, or rather if the dysbiosis induced during stress causes altered intestinal physiology.

We found that the level of kynurenine is increased after chronic stress, in a manner dependent on *Lactobacillus* levels. Kynurenine can readily cross the blood-brain barrier to drive depression within the CNS by disrupting neurotransmitter balance and driving neuroinflammation^27^,^47^. A recent study by Agudelo *et al*.^26^ identified this pathway as also being disrupted in stressed mice using the same model of UCMS. Taken together, these new findings point to disruptions in tryptophan-kynurenine metabolism as an important factor in mediating despair behavior. IDO1 is the main enzyme responsible for conversion of tryptophan to kynurenine outside of the liver, and its expression and activity can be directly inhibited by reactive oxygen species (ROS)^32^. Members of the *Lactobacillus* family have the capacity to produce high levels of ROS, as a means of maintaining their niche^36^,^37^. In our study, we have shown that decreased levels of ROS in stressed animals correlate with an increase in intestinal *ido1* transcripts, thus potentially explaining our observed increase in circulating kynurenine. Moreover, several studies have shown that inhibiting IDO1 activity (such as with the small molecule 1-methyl tryptophan) has potent effects in ameliorating depressive-like behaviors both in chronic stress and inflammation-induced sickness behavior models^48^,^49^,^50^.

The inhibition of IDO1 by *Lactobacillus-*derived ROS is likely just one of the mechanisms through which *Lactobacilli*, and *L. reuteri* in particular, contribute to host physiology and modulate behavior. Our findings are in accordance with the previously reported beneficial effect of *L. reuteri* administration on despair and anxiety-like behaviors^25^. Nevertheless, in their study, Bravo and colleagues have shown that *L. reuteri* can modulate GABA receptor expression in the CNS, via the vagus nerve^25^. The vagus nerve has been shown to carry peripheral signals and modulate inflammatory and stress reponses^51^,^52^,^53^,^54^. Whether the two results are connected remains to be investigated. It is possible that intestinal kynurenine can signal on the afferent vagal terminals and modulate its effects in the CNS, including its modulation of the hypothalamus-pituitary adrenal (HPA) axis. To this point, it is important to consider the contribution of liver TDO to peripheral kynurenine levels. TDO expression and activity are increased by glucocorticoids and in response to acute stress, and play a role in glucocorticoid levels homeostasis^55^,^56^,^57^. Whether TDO levels decrease chronically in our long-term stress model (following an expected decrease of corticosterone) or what effect the *Lactobacillus* administration has remains to be investigated. We have also considered other possible mechanisms for the behavioral effects of *L. reuteri* supplementation, mediated by the immune system, or other populations of commensals affected by the treatment. Further studies will be necessary to assess the chronological and the hierarchical role of each pathway during despair behavior development, as well as how these pathways affect the CNS.

Altogether, our results indicate that the microbiome can play a causative role in the development and symptomatology of depression. Further studies are needed to prove a causal relationship between intestinal *Lactobacillus* levels and depressive-like behavior. Moreover, investigation of whether *Lactobacilli* can play a similar function in human biology and if manipulation of *Lactobacillus* levels and/or local induction of ROS production in the gut could be used to treat psychiatric disorders is warranted.

## Methods and Materials

### Animals

All methods were performed in accordance with the relevant guidelines and regulations of the University of Virginia and approved by the University of Virginia Animal Care and Use Committee. Male C57BL/6 and BALB/c (8 weeks old) were purchased from Jackson or Taconic laboratories as described in the text. The mice were maintained on a 12 hours light/dark cycle with lights on at 7am. Chronic stress was started after at least 1 week of acclimation. All behavioral interventions were performed between 4 pm and 7 pm and take-downs were performed between 11am and 2 pm. Sample size was selected to be similar to previously reported behavioral experiments. Animals were housed 2–3 per cage and cages were randomly assigned to control or experimental groups. In each experiment, every group consisted of at least 3 cages in order to minimize cage effects. Investigators were not blinded to the group allocation. Experiments were conducted in such a way as to make sure that all experimental groups were exposed to the same environments. Animals were excluded from the experiments if they developed illnesses (e.g. dermatitis) that might affect the outcome of the results. Certain measurements are occasionally not available for each mouse enrolled in the study due to technical issues (e.g. fecal sample not provided, sample loss).

### Chronic mild stress protocol

Mice were subjected daily to an acute stressor (1–2 hours: restraint, loud white noise, crowded housing, strobe light) and an overnight stressor (12–24 hours: 45° cage tilting, repeated cage changes, wet bedding, dark deprivation) presented in a randomized fashion as described in the literature^13^. For restraint stress, mice were placed in clean 50 mL conical tubes with pierced holes for ventilation for 1 hour. For crowded housing stress, mice were placed on top of the cage wire for 2 hours, after which their cage was changed. For wet bedding stress, 200 mL of water was added to the bedding of a clean cage. All procedures used autoclaved, sterile materials (bedding, water) in order to prevent contamination. Food, water intake, and weights were monitored in initial experiments and no major changes were observed in the stressed group. For subsequent experiments, monitored parameters are indicated on matching experimental design figures. Sequencing results showed no new OTUs in the stressed microbiota, indicating the lack of contamination during the stress protocol.

### Behavioral assessment

Despair behavior was assessed at time points of interest using the forced swim test, as described in the literature^58^. The last 4 minutes (out of 6 minutes total test) were scored for escape behavior, defined as active swimming, with all four limbs and tail moving. The test was conducted using autoclaved water and disinfected containers. Anxiety/compulsive behavior was assessed using the nestlet-shredding test^16^. The mice were singled housed 30–60 minutes before the beginning of the test. Each mouse then received a pre-weighed nestlet and allowed undisturbed activity for either 30 or 60 minutes. At the end of the test, the piece of nestlet that was still intact was weighed.

### DNA isolation

Whole genomic DNA was isolated via phenol-chloroform extraction. Briefly, a fecal pellet was placed in a 2 mL tube containing 200 μL silica-zirconia beads (0.1 mm). The tube was filled with 750 μL extraction buffer, 200 μL 20% SDS and 750 μL phenol-chloroform-isoamyl alcohol (25:24:1). After disruption, the aqueous phase was separated by centrifugation and cleaned up with two washes of chloroform-isoamyl alcohol (24:1). The DNA was precipitated and resuspended in 10 mM Tris solution.

### Fecal microbiota sequencing

For 16S rRNA sequencing, the V3-V4 region of the 16S rRNA gene was amplified for 25 cycles using specific primers with adapter overhangs as per the Illumina library preparation guide (Sup. Table 1). Following purification of the PCR products, individual indexes were added to the amplicons by PCR. The amplicons were purified, pooled in equal quantities, and then sequenced on the Illumina MiSeq platform. Reads with an average quality score below 25 (from any 10-bp window) or a mismatched barcode were removed. Paired-end reads were then merged using the software FLASH^59^. Merged reads were analyzed using the QIIME pipeline with default parameters to remove chimeric, pick 97%-identity OTUs and assign taxonomy^60^.

### Selective culture of *Lactobacilli* and *L. reuteri* preparation

Fecal pellets were resuspended in 1 mL of deMan, Rogosa and Sharpe (MRS) broth supplemented with sodium azide (0.02% w/v) to select *Lactobacillus*^19^. After brief decanting of insoluble fecal material, samples were further diluted 1:1000 in MRS/azide and 50 μL of this dilution were spread on MRS/azide agar plates and grown overnight at 37 °C, in aerobic conditions. The plates were then imaged with a Bio-Rad gel imager and colonies were counted using the “particle counter” plugin in ImageJ.

For *Lactobacillus* supplementation experiments, L. *reuteri* was obtained from ATCC (23272) and cultured aerobically according to manufacturer instructions. Radiated food pellets were pulverized in a blender and kneaded with fresh *L. reuteri* (2 billion CFU/mouse/day) and water, or with MRS culture broth and water for control. The animals received fresh food prepared daily.

### Intestinal transit time measurement

Large intestinal transit time was measured as previously described^61^. Briefly, mice were anesthetized with isofluorane and a 3 mm diameter glass bead was inserted 2 cm inside the rectum with a lubricated rod. The mice were then placed in an empty cage without bedding and the time to bead expulsion was measured.

### Intestinal tissue analysis

The small intestine was dissected by excising under the stomach and before the cecum. Mesenteric fat and Peyer’s patches were carefully removed using fine forceps. The intestine was opened longitudinally and the contents were removed in two PBS washes. Excess liquid was gently absorbed using kimwipes and the tissue was weighed. Intestinal cells were then isolated as previously described^62^. Briefly, the mucus layer and epithelial cells were shook off in two washes with HBSS/5% FBS/2 mM EDTA. The tissue was then digested with collagenase VIII (Sigma, C2139) for 12–15 minutes, filtered, and the cells washed twice with HBSS/5% FBS/2 mM EDTA and finally resuspended in FACS buffer (0.01 M PBS, 1% BSA, 2 mM EDTA, 0.1% sodium azide). Cell counts and viability were determined using an acridine orange/propidium iodine assay on a Nexcelom cell counter.

### Metabolite analysis

Blood was collected by cardiac puncture and centrifuged at 10,000 × g for 3 minutes in gel tubes for serum preparation. Frozen (−80 °C) serum samples were shipped to the University of Michigan Metabolomics Core for untargeted metabolomics analysis. Metabolites isolated by positive and negative ion selection were analyzed by mass spectrometry. Mass spectrometry peak intensities were further analyzed using the MetaboAnalyst online software. Peak values were filtered using the interquantile range, normalized to the group sum, then log transformed and auto-scaled for principal component analysis and further statistical tests^63^. Identifiable significant metabolites were analyzed through pathway analysis, followed by further manual pathway enrichment.

### ROS quantification

Fresh fecal samples were collected in sterile 2 mL tubes, weighed, and resuspended in 1 mL sterile PBS. After brief sedimentation of insoluble particles, 500 μL of bacterial slurry were incubated at 37 °C for 30 minutes. Following bacterial culture centrifugation, 50 μL of supernatant was reacted using the Amplex Red hydrogen peroxide/peroxidase assay kit (Thermo Fisher, cat. A22188) according to manufacturer’s protocol. For ROS production by individual *Lactobacillus* species, fecal *Lactobacilli* were cultured as described above for 20 hours. Individual colonies were selected and dissociated in 200 uL MRS media and incubated for 30 minutes at 37 °C. The resulting ROS concentration was measured as described above. The identity of each colony was verified using specific primers (Sup. Table 1).

### qPCR

For RNA quantification, frozen tissues (brain and intestine) were homogenized by bead beating in RNA TRI Reagent (Life technologies) and RNA was extracted according to manufacturer’s protocol. cDNA was synthethized with the High Capacity cDNA Reverse Transcription kit (Life Technologies). cDNA was amplified using the Sensifast Sybr NO-ROX kit (Bioline), according to manufacturer’s instructions. Gapdh was measured as a normalizer for each sample. Results were analyzed by the relative quantity (ΔΔCt) method^64^.

For total 16S rRNA quantification, we followed the BactQuant protocol described by Liu *et al*.^65^. Briefly, the 16S gene was amplified using primers directed to the V3-V4 rRNA region (Sup. Table 1). *L. reuteri* DNA was used for the standard curve. For relative quantification of total *Lactobacillus* or specific *Lactobacillus* species, the ΔΔCt method was used to compare *Lactobacillus*-specific amplification to that of the 16S rRNA gene. Reactions were performed using the Sensifast Sybr NoROX kit from Bioline (BIO-98005). Primer sequences are available in Sup. Table 1.

### Statistical analysis

All statistical analyses were performed in Prism. The results of the statistical tests are presented within the results section. Analyses involving two groups were performed using a two-tailed t-test. If the variances between groups were significantly different, a Welch’s correction was applied. For experiments involving stress and another variable (e.g. *L.reuteri* treatment), data were analyzed with a two-way ANOVA. For the metabolomics experiments involving only 3 groups, a one-way ANOVA was utilized. Outliers were excluded if they fell more than two standard deviations from the mean. For all analyses, the threshold for significance was at p < 0.05. Repeats for each experiment, if performed, are specified in the figure legend corresponding to the respective panel.

## Additional Information

**How to cite this article:** Marin, I. A. *et al*. Microbiota alteration is associated with the development of stress-induced despair behavior. *Sci. Rep.* **7**, 43859; doi: 10.1038/srep43859 (2017).

**Publisher's note:** Springer Nature remains neutral with regard to jurisdictional claims in published maps and institutional affiliations.

## Supplementary Material

Supplementary Figures
