# Supplementary material for: Microbiota alteration is associated with the development of stress-induced despair behavior
Source: Sci Rep. 2017 Mar 7;7:43859. doi: 10.1038/srep43859 (PMC5339726; doi:10.1038/srep43859)
Supplement: Supplementary Figures [file srep43859-s1.pdf]

# Microbiota alteration is associated with the development of stress-induced despair behavior

Ioana A. Marin[1,2,3], Jennifer E. Goertz[1,3], Tiantian Ren[4], Stephen S. Rich[5], Suna Onengut-Gumuscu[5], Emily Farber[5], Martin Wu[4], Christopher C. Overall[1,2], Jonathan Kipnis[1,2,3#*] and Alban Gaultier[1,2,3#*]

[1]Center for Brain Immunology and Glia, [2]Department of Neuroscience, [3]Graduate Program in Neuroscience, School of Medicine, University of Virginia, Charlottesville, VA 22908, USA, [4]Department of Biology, University of Virginia, Charlottesville, VA 22904, USA, [5]Center for Public Health Genomics, University of Virginia, Charlottesville, VA 22908.

#Equally contributing senior authors

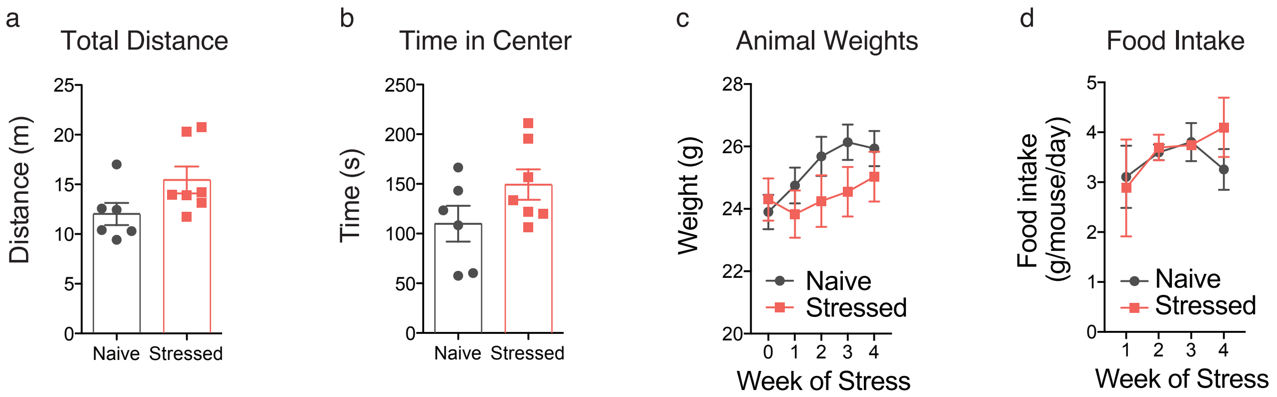


**Supplementary Figure 1**. Unpredictable chronic mild stress (UCMS) does not affect general activity. (a) Total distance transversed during the open field test (n = 6 naïve and 7 stressed; mean ± s.e.m). (b) Time spent in the center of the field during a 15 minute test (n = 6 naïve and 7 stressed; mean ± s.e.m). (c) Animal weights (n = 6 mice per group; 2-way ANOVA, no significant effect of stress; mean ± s.e.m). (d) Mean food intake (n = 4 per cage measurements, 2-way ANOVA, no significant effect of stress; mean ± s.e.m).

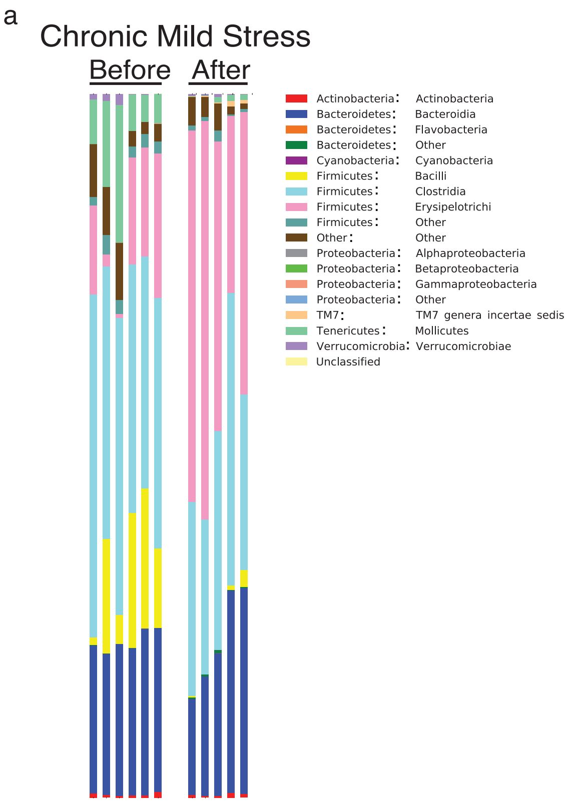


b

| Taxon | Naive mean | Stressed mean | p value |
|---|---|---|---|
| *Turicibacteraceae* genus | 1.232E-01 | 2.171E-02 | 1.662E-07 |
| *Coprococcus* | 2.008E-02 | 5.193E-03 | 1.984E-07 |
| *Anaeroplasma* | 6.168E-02 | 8.570E-03 | 3.214E-07 |
| *Lactobacillus* | 1.259E-01 | 1.589E-02 | 7.529E-07 |
| *Mollicutes* genus | 1.282E-02 | 3.659E-03 | 8.004E-06 |
| *Allobaculum* | 5.957E-04 | 3.068E-01 | 2.356E-05 |
| *Clostridium* | 7.665E-03 | 5.571E-02 | 3.049E-05 |
| *Bifidobacterium* | 9.382E-06 | 6.614E-04 | 6.743E-05 |
| *Peptococcaceae* genus | 6.047E-03 | 2.026E-03 | 3.270E-04 |
| *Ruminococcus* | 4.452E-03 | 2.734E-02 | 6.372E-04 |
| *Eubacterium* | 7.271E-04 | 2.674E-04 | 8.831E-04 |
| TM7 | 0.000E+00 | 4.503E-03 | 2.106E-03 |
| *Clostridiales* genus | 6.970E-02 | 3.802E-02 | 3.294E-03 |
| *Coprobacillus* | 9.335E-04 | 0.000E+00 | 6.795E-03 |
| *Lachnospiraceae* genus | 1.074E-03 | 3.180E-03 | 1.117E-02 |
| *Erysipelotrichaceae* genus | 1.623E-03 | 3.701E-03 | 1.196E-02 |
| *Clostridiaceae* genus | 0.000E+00 | 3.800E-04 | 1.681E-02 |
| *Coriobacteriales* genus | 3.753E-05 | 0.000E+00 | 1.843E-02 |
| *Cyanobacteria* genus | 4.691E-06 | 1.784E-02 | 2.553E-02 |
| *Anaerofustis* | 2.345E-05 | 1.970E-04 | 3.213E-02 |
| *Lachnobacterium* | 0.000E+00 | 3.518E-04 | 3.542E-02 |
| *Actinobacteria* genus | 0.000E+00 | 1.126E-04 | 3.793E-02 |
| *Catabacteriaceae* genus | 1.293E-02 | 7.064E-03 | 4.177E-02 |
| *Slackia* | 4.691E-05 | 0.000E+00 | 4.815E-02 |

**Supplementary Figure 2**. Unpredictable chronic mild stress (UCMS) induces microbiota dysregulation. (a) Representative graphs of bacterial class distribution in individual subjects show a decrease in bacilli (yellow) in a different sequencing co-hort (n = 6 naïve and 5 stressed). (b) List of significant genera between naive and stressed samples.

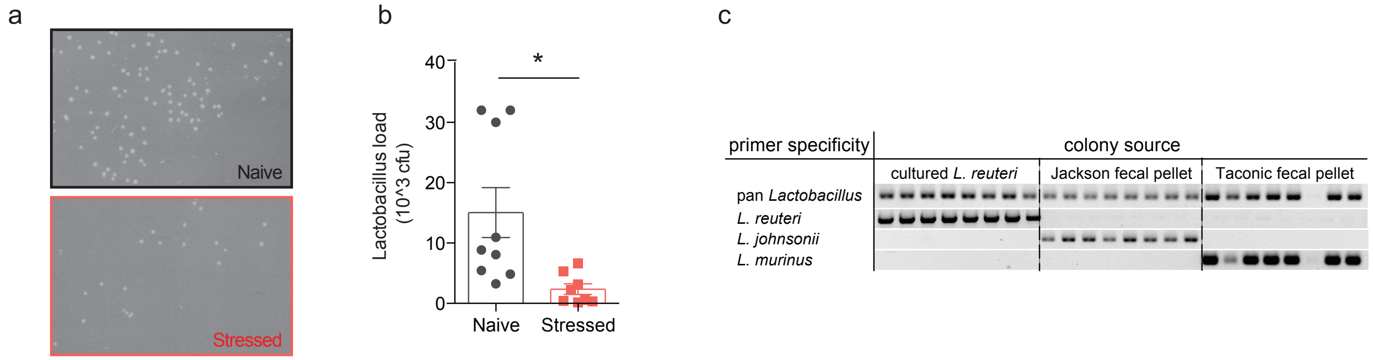


**Supplementary Figure 3**. Quantification of *Lactobacillus* by selective culture. (a) Representative images of *Lactobacillus* colonies from naive and stressed mice. (b) Quantification of colonies obtained from fecal samples of naive and stressed mice (n = 9 naïve and 8 stressed, representative of 2 independent experiments; two-tailed t-test with Welch's correction, *p<0.05; mean ± s.e.m). (c) PCR verification of *Lactobacillus* colonies obtained by selective culture from fecal samples, with *L.reuteri* colonies as controls.

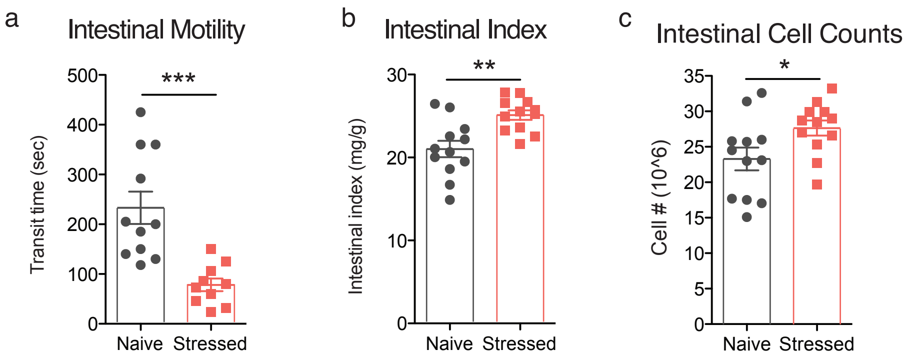


**Supplementary Figure 4**. Stressed mice show signs of altered intestinal physiology. (a) Intestinal motility of naive and stressed mice (n = 11 naive and 10 stressed mice per group; two-tailed t-test with Welch's correction; ***p < 0.001; mean ± s.e.m). (b) Small intestine index, relative to body weight (n = 12 mice per group, two-tailed t-test; **p < 0.01; mean ± s.e.m). (c) Total intestinal cell numbers (n = 12 mice per group, two-tailed t-test; *p < 0.05; mean ± s.e.m).

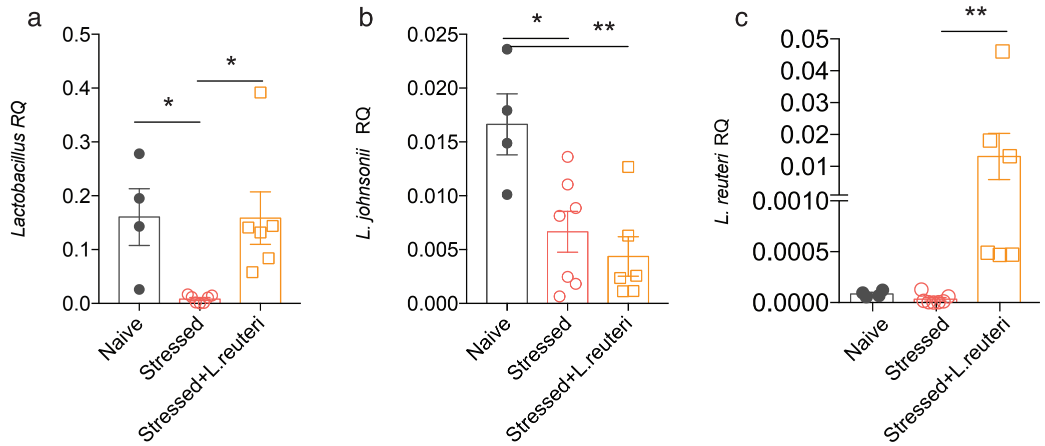


**Supplementary Figure 5**. Quantification of *Lactobacillus* species. (a) Quantification of total fecal *Lactobacillus* levels after stress and *L. reuteri* supplementation (n = 4-6 animals per group; one-way ANOVA with Tuckey's multiple comparisons, *p < 0.05; mean ± s.e.m). (b) Quantification of fecal *L. johnsonii* levels after stress and *L. reuteri* supplementation (n = 4-6 animals per group; one-way ANOVA with Tuckey's multiple comparisons, *p < 0.05, **p<0.01; mean ± s.e.m). (a) Quantification of fecal *L. reuteri* levels after stress and *L. reuteri* supplementation (n = 4-6 animals per group; Kruskal-Wallis test with Dunn's multiple comparisons, **p < 0.01; mean ± s.e.m).

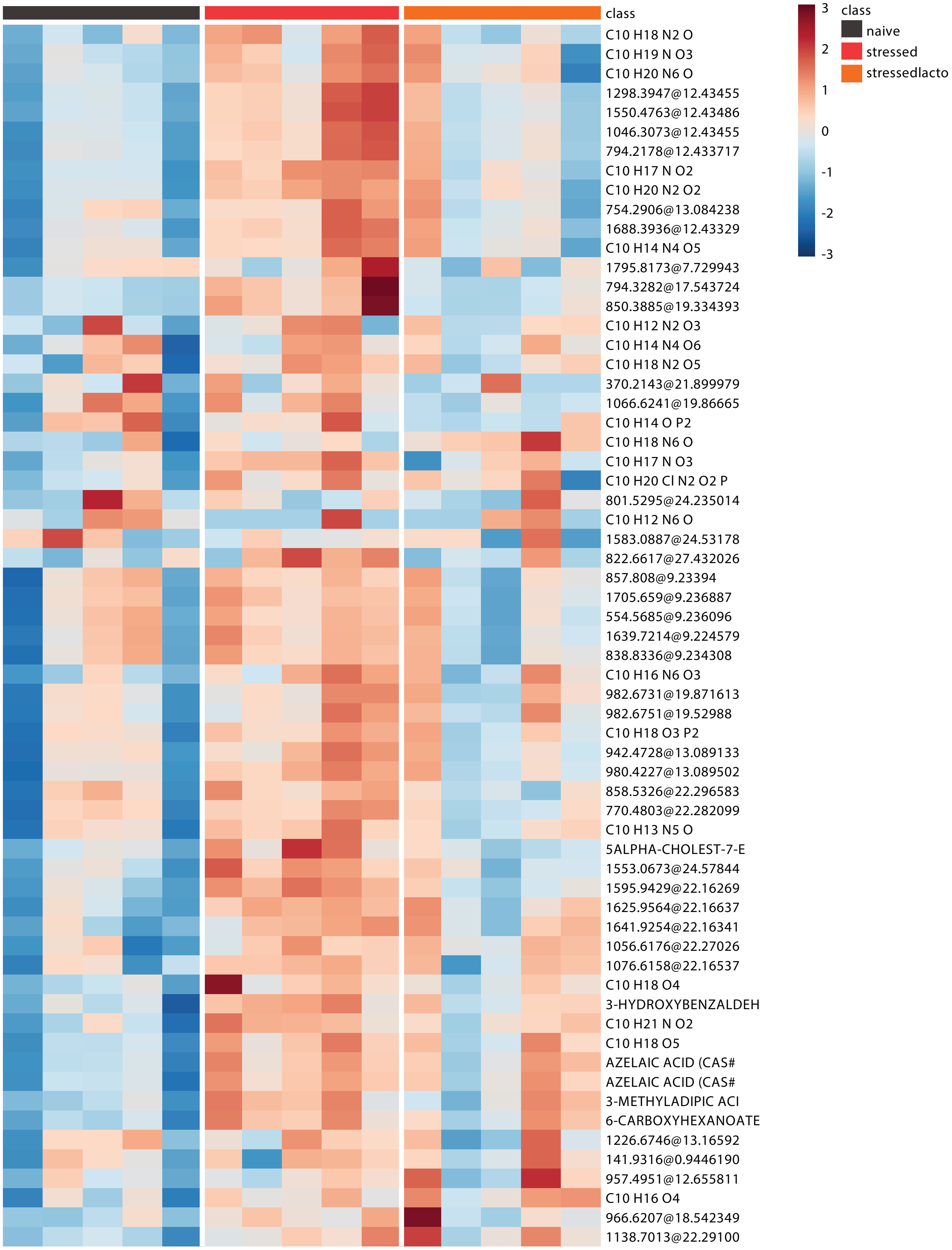


**Supplementary Figure 6**: Representative heatmap of top significant metabolites
Top 63 most significant metabolites represented using heat-map analysis.

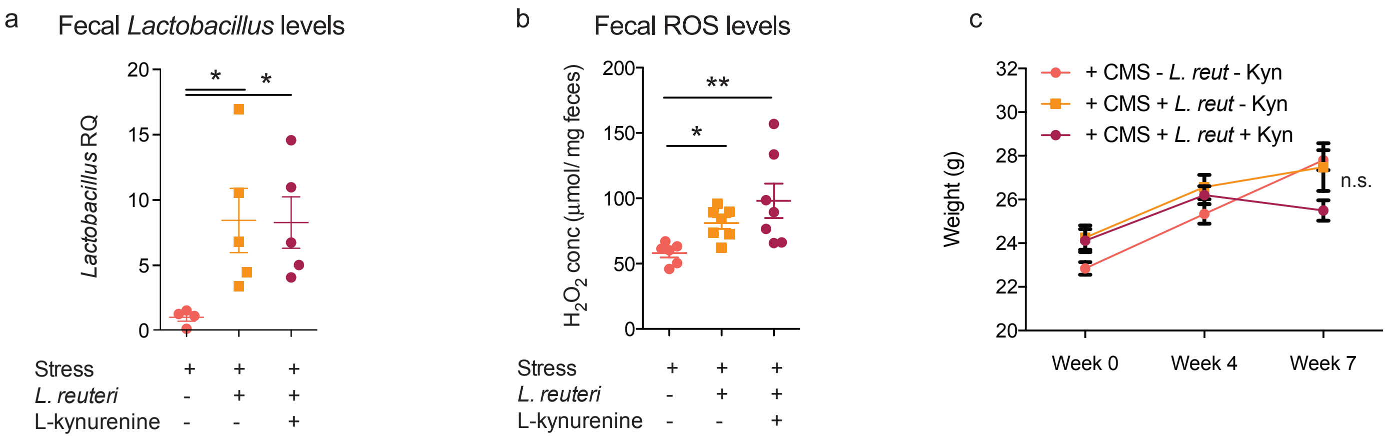


**Supplementary Figure 7**. *Lactobacillus* treatment elevates fecal *Lactobacillus* and ROS levels regardless of kynurenine levels. (a) Quantification of fecal *Lactobacillus* levels after *L. reuteri* supplementation and kynurenine treatment (n = 4-5 animals per group; modified one-way ANOVA with Dunn's multiple comparisons, *$p < 0.05$; mean ± s.e.m) (b) Quantification of fecal $H_2O_2$ levels after *L. reuteri* supplementation and kynurenine treatment (n = 6-7 animals per group; modified one-way ANOVA with Dunn's multiple comparisons, *$p < 0.05$, **$p < 0.001$; mean ± s.e.m). (c) Animal weights at key treatment timepoints (n = 6-7 animals per group, 2-way ANOVA, no significant effect of treatment; mean ± s.e.m).

| Name | Forward seq | Reverse seq |
|---|---|---|
| 16S for library with adapters (per Illumina guide) | TCGTCGGCAGCGTCAGATGTGTATAAGAGACAGCCTACGGGNGGCWGCAG | GTCTCGTGGGCTCGGAGATGTGTATAAGAGACAGGACTACHVGGGTATCTAATCC |
| 16S [1] | CCTACGGGDGGCWGCA | GGACTACHVGGGTMTCTAATC |
| *Lactobacillus* [2] | TGGATGCCTTGGCACTAGGA | AAATCTCCGGATCAAAGCTTACTTAT |
| SFB [3] | GACGCTGAGGCATGAGAGCAT | GACGGCACGGATTGTTATTCA |
| *L. reuteri* [4] | ACCTGATTGACGATGGATCACCAGT | CCACCTTCCTCCGGTTTGTCA |
| *L. johnsonii* [5] | GAGCTTGCCTAGATGATTTTA | ACTACCAGGGTATCTAATCC |
| *L. murinus* [6] | GCAATGATGCGTAGCCGAAC | GCACTTTCTTCTCTAACAACAGGG |
| ido1v1 [7] | TGAAGATGTGGGCTTTGCTCT | TGTGGGCAGCTTTTCAACTTC |

**Supplementary Table 1**. Primer sequences
List of primer sequences used in the study.

SUPPLEMENTAL REFERENCES

1. Liu CM, Aziz M, Kachur S, Hsueh PR, Huang YT, Keim P et al. BactQuant: an enhanced broad-coverage bacterial quantitative real-time PCR assay. BMC microbiology 2012; 12: 56.

2. Haarman M, Knol J. Quantitative real-time PCR analysis of fecal *Lactobacillus* species in infants receiving a prebiotic infant formula. Appl Environ Microb 2006; 72(4): 2359-2365.

3. Ivanov, II, Atarashi K, Manel N, Brodie EL, Shima T, Karaoz U et al. Induction of intestinal Th17 cells by segmented filamentous bacteria. Cell 2009; 139(3): 485-498.

4. Kwon HS, Yang EH, Yeon SW, Kang BH, Kim TY. Rapid identification of probiotic *Lactobacillus* species by multiplex PCR using species-specific primers based on the region extending from 16S rRNA through 23S rRNA. Fems Microbiol Lett 2004; 239(2): 267-275.

5. Walter J, Tannock GW, Tilsala-Timisjarvi A, Rodtong S,Loach DM, Munro K, Alatossava T. Detection and Identification of Gastrointestinal Lactobacillus Species by Using Denaturing Gradient Gel Electrophoresis and Species-Specific PCR Primers. Appl Environ Microbiol. 2000; 66(1): 297-303.

6. Harley IT, Giles DA, Pfluger PT, Burgess SL, Walters S, Hembree J, Raver C, Rewerts CL, Downey J, Flick LM, Stankiewicz TE, McAlees JW, Wills-Karp M, Balfour Sartor R, Divanovic S, Tschöp MH, Karp CL. Differential colonization with segmented filamentous bacteria and Lactobacillus murinus do not drive divergent development of diet-induced obesity in C57BL/6 mice. Mol Metab 2013. 10;2(3):171-83.

7. Agudelo LZ, Femenia T, Orhan F, Porsmyr-Palmertz M, Goiny M, Martinez-Redondo V et al. Skeletal muscle PGC-1alpha1 modulates kynurenine metabolism and mediates resilience to stress-induced depression. Cell 2014; 159(1): 33-45.
